# Alpha-synuclein pathology of olfactory bulbs/peduncles in the Vantaa85+ cohort exhibit two divergent patterns: a population-based study

**DOI:** 10.1007/s00401-021-02364-6

**Published:** 2021-09-01

**Authors:** Eloise H. Kok, Sara Savola, Anna Raunio, Minna Oinas, Jarno Tuimala, Tuomo Polvikoski, Mia Kero, Karri Kaivola, Pentti J. Tienari, Anders Paetau, Liisa Myllykangas

**Affiliations:** 1 Department of Pathology, University of Helsinki, HUS Diagnostic Center, Helsinki University Hospital, P.O. Box 21, 00014 Helsinki, Finland; 2 Department of Pathology, University of Helsinki, P.O. Box 21, 00014 Helsinki, Finland; 3 Division of Clinical Neuroscience and Rehabilitation, Department of Neurosurgery, Ophthalmology and Otorhinolaryngology, University Hospital of North-Norway, 9038 Tromso, Norway; 4 Institute of Neuroscience, Newcastle University, Newcastle upon Tyne, NE2 4HH UK; 5 Translational Immunology, Research Programs Unit, University of Helsinki, P.O.Box 63, 00014 Helsinki, Finland; 6 Department of Neurology, Helsinki University Hospital, P.O. Box 63, 00014 Helsinki, Finland

In Lewy body diseases, including Parkinson’s disease (PD) and dementia with Lewy bodies (DLB), alpha-synuclein accumulates intraneuronally in the central and peripheral nervous systems [1, 3, 8]. Recent studies [3, 6] have suggested that spread of alpha-synuclein positive Lewy-related pathology (LRP) follows two distinct patterns—caudo-rostral progression and an amygdala-based progression, the latter usually seen together with Alzheimer’s disease (AD) pathology [6].

The olfactory system is affected in almost all PD and DLB cases. However, distribution and possible spreading routes of olfactory pathology may differ between PD and DLB [2]. In this context, a hospital-based neuropathological study indicated that LRP in olfactory bulb (OB) can be identified in two anatomically different areas: anterior olfactory nucleus (referred as AON) and periphery of the OB (referred to as peripheral) [7]. AON pathology was strongly associated with a high burden of LRP in amygdala [7].

We have previously investigated the brain and spinal cord LRP in a population-based sample [6]. Here, we analyzed LRP in the olfactory regions (OB and olfactory peduncle, OP) to complement our previous study and to test how this LRP pathology correlates with LRP patterns in the brain.

Materials and methods have been described in detail previously [6] (see also online resource). Briefly, OB/OP samples of Finns aged 85 or over (Vantaa 85 + cohort) were collected, fixed in formalin and embedded in paraffin. Sections of the samples were stained using alpha-synuclein antibody 5G4. Two assessors blinded to clinical, genetic and neuropathological information screened AON and peripheral regions and graded LRP semi-quantitatively based on the grading system used by Sengoku et al. [7] and DLB Consortium guidelines [4]. The highest two grades (3–4) were merged to retain statistical power.

The Vantaa 85 + study’s neuropathological cohort included 291 cases with OB/OP samples. 119 (41%) were scored positive for alpha-synuclein immunohistochemical staining. Of our positive OB/OP cases, 110 (92%) cases were positive in both AON and peripheral and 9 (8%) only in peripheral. No cases were AON-only positive. Based on staining intensity, we classified 79 cases (66%) as AON predominant and 21 cases (18%) as peripherally predominant. The predominance was unclear in 19 (16%) cases. In general, there was more staining in AON than periphery of olfactory regions. Table 1 reveals the alpha-synuclein positivity in OB/OP samples grouped according to DLB Consortium classifications [5] and LRP progression-based categorisation [6]. Figure 1 displays how OB/OP predominance relates to caudo-rostral and amygdala-based LRP progression type. Further results are shown in the online resource.

**Table 1:** Characteristics of the Vantaa 85 + Study and Olfactory bulb/peduncle Lewy-related (LRP) pathology categorised by DLB Consortium classification [5] and LRP progression-based classification [6]

	No LRP [6]	LRP detected						
		DLB consortium classification [5] *n* = 119					LRP progression-based [6] *n* = 118	
		Non-classifiable	Brainstem	Amygdala predominant	Limbic	Diffuse Neocortical	Caudo-rostral	Amygdala-based
	*n* = 172	*n* = 11	*n* = 17	*n* = 10	*n* = 40	*n* = 41	*n* = 80	*n* = 38
Women %	85	82	84	90	80	74	76	87
Mean age at death (years)	92.3	91.3	93.8	93.0	92.3	92.2	92.6	92.3
Periphery LRP (*n*, %)								
None	157 (91.3)	11 (100)	4 (23.5)	0 (0)	0 (0)	0 (0)	15 (18.8)	0 (0)
Sparse positivity	13 (7.6)	0 (0)	9 (52.9)	7 (70.0)	34 (85.0)	29 (70.7)	51 (63.7)	27 (71.1)
Scattered positivity^a^	2 (1.2)	0 (0)	4 (23.5)	3 (30.0)	6 (15.0)	12 (29.3)	14 (17.5)	11 (28.9)
Dense positivity^a^	0 (0)	0 (0)	0 (0)	0 (0)	0 (0)	0 (0)	0 (0)	0 (0)
AON LRP (*n*, %)								
None	163 (94.8)	11 (100)	5 (29.4)	0 (0)	2 (5.0)	0 (0)	18 (22.5)	0 (0)
Sparse positivity	9 (5.2)	0 (0)	9 (52.9)	3 (30.0)	9 (22.5)	0 (0)	17 (21.3)	4 (10.5)
Scattered positivity^a^	0 (0)	0 (0)	3 (17.6)	4 (40.0)	12 (30.0)	3 (7.3)	13 (16.3)	9 (23.7)
Dense positivity^a^	0 (0)	0 (0)	0 (0)	3 (30.0)	17 (42.5)	38 (92.7)	32 (40.0)	25 (65.8)
OB/OP LRP predominance (*n*, %)^b^								
Peripheral	13 (86.7)	0 (0)	5 (38.5)	1 (10.0)	2 (5.0)	0 (0)	6 (9.2)	2 (5.3)
AON	0 (0)	0 (0)	2 (15.4)	7 (70.0)	29 (72.5)	41 (100)	44 (67.7)	34 (89.5)
Unclassifiable	2 (13.3)	0 (0)	6 (46.2)	2 (20.0)	9 (22.5)	0 (0)	15 (23.1)	2 (5.3)

^a^Scattered = 1–3 LB at 100 × magnification; dense ≥ 4 LB at 100 × magnification, according to [7]. LN were measured visually. Dense groups were combined to retain statistical significance. *AON*  anterior olfactory nucleus, *LRP* Lewy-related pathology, *OB/OP* Olfactory bulb/Olfactory peduncle. Case numbers of DLB Consortium classification and LRP progression-based differ due to one case being omitted from analyses in [6] for having extremely high alpha-synuclein pathology. ^b^The unclassifiable category doesn't include samples that had no peripheral and no AON LRP

**Fig. 1:**
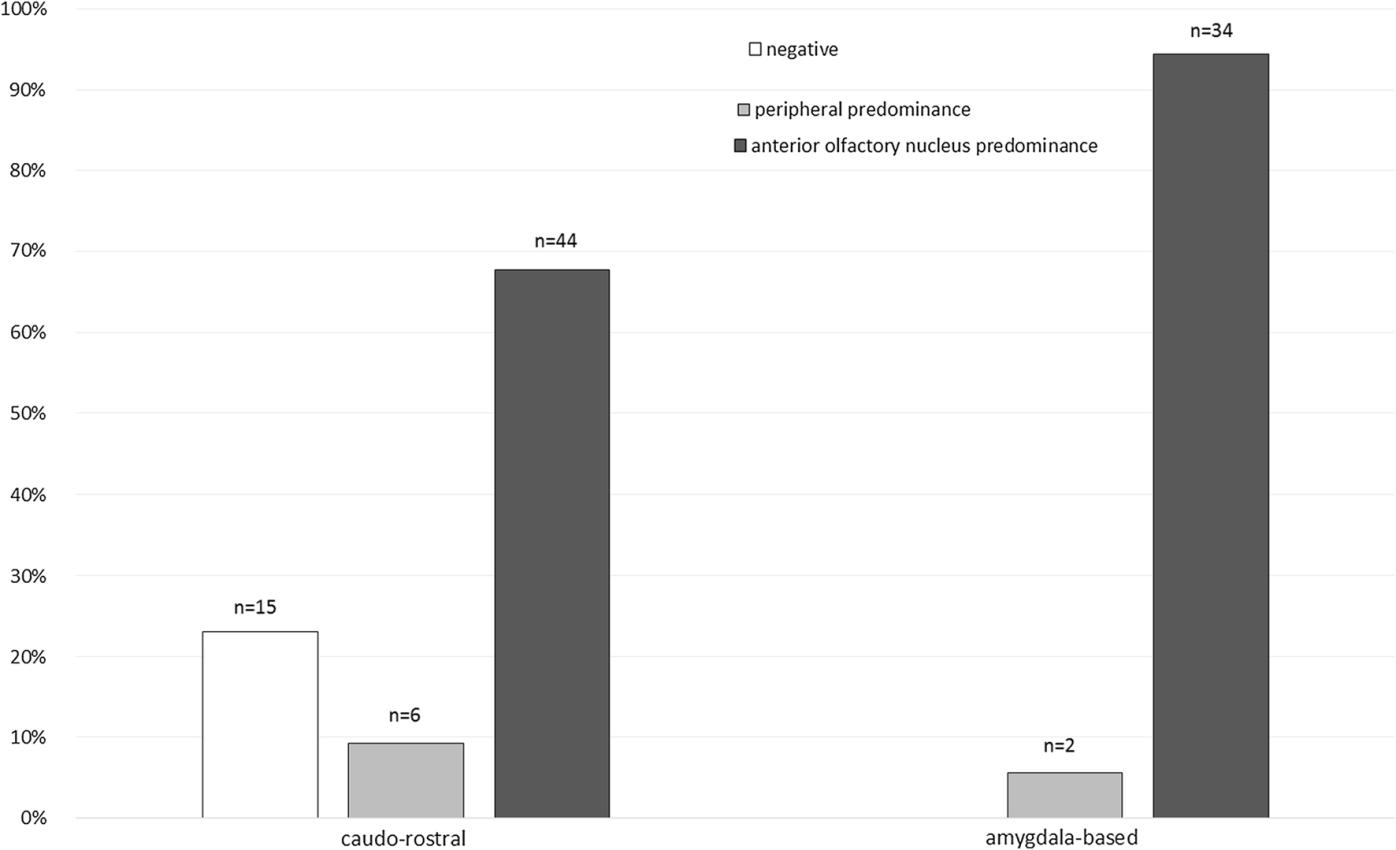
Olfactory bulb/peduncle alpha-synuclein staining predominance categorised by LRP progression-based classification [6]. Percentages add up to 100% within each LRP progression-based classification category, meaning all the caudo-rostral cases equal 100% spread across negative, peripheral and anterior olfactory nucleus predominance for alpha-synuclein staining. Similarly, the amygdala-based category cases add up to 100% spread across the peripheral and anterior olfactory nucleus predominance for alpha-synuclein staining (there were no amygdala-based cases that were negative in olfactory bulb/peduncle alpha-synuclein staining). When comparing these groups, age and sex adjusted *p* value < 0.001

When considering the DLB Consortium classification, our results suggest that strong AON positivity is associated with strong DLB pathology, particularly neocortical DLB (Table 1, online resource Fig. 1 and Table 1, *p* < 0.001). All amygdala-predominant cases (DLB Consortium classification) were OB/OP positive. Brainstem-predominant DLB cases had no or mild positivity for OB/OP pathology (Table 1, online resource Table 1). All non-classifiable cases (DLB Consortium classification with minimal medulla pathology) were negative for OB/OP pathology. Generally, cases with mild or no LRP pathology or pathology only in the brainstem tended to be negative for OB/OP pathology, whereas cases with strong LRP throughout the brain also had strong AON and peripheral OB/OP pathology (mostly with AON predominance, Fig. 1, online resource).

When considering the dichotomous (caudo-rostral or amygdala-based) progression-based classification [6], the predominance of OB/OP alpha-synuclein patterns were pronounced (Fig. 1, *p* < 0.001). Amygdala-based cases tended to have AON predominance compared to caudo-rostral cases. None of the amygdala-based cases were negative for OB/OP pathology, whereas 23% of caudo-rostral cases were negative (Fig. 1, Table 1). In general, caudo-rostral cases showed milder OB/OP pathology (both peripheral and AON) (Table 1). K-means clustering of our OB/OP data and with other measured brain regions showed similar grouping (Fig. 2, online resource), in concordance with our previous results [6].

Interestingly, there were 15 cases (13%) positive for LRP only in OB/OP (not in brain or brainstem). These had modest AON and peripheral LRP pathology (see Table 2, online resource). On closer inspection, these cases had variable AD-type pathology, and in most cases (13, except two indeterminable) had peripherally predominant OB/OP LRP. A possible explanation could be early stage alpha-synuclein accumulation in these cases. This would be consistent with the previous study by Sengoku et al. suggesting that the OB/OP pathology begins in the periphery, and later proceeds to the anterior olfactory nucleus [7]. This interpretation is also consistent with the finding that there were no cases in our study cohort that were positive only in the anterior olfactory nucleus.

Substantia nigra neuron loss was significantly associated with OB/OP staining (see Fig. 3, online resource, *p* < 0.001). AD pathology according to Braak staging was significantly associated with OB/OP staining patterns (see Table 1, online resource, *p* = 0.002 comparing none versus all positive OB/OP staining), but not with CERAD scores. OB/OP staining predominance showed a trend towards association with moderate or frequent CERAD score but was not significant (82% AON predominance versus 62% peripheral, *p* = 0.07). Consistent with the AD pathology results, analysis by OB/OP predominance showed *APOE* ε4 was found in 49% of the AON predominant cases versus 26% of peripheral cases but was not significant after age and sex correction (*p* = 0.10). The number of cases with dementia when comparing OB/OP staining predominance were 66 (83.5%) subjects with the AON predominance versus 12 (57%) cases in the peripheral predominance group (*p* = 0.006).

Whilst these results are from a post-mortem study that has a relatively small number of cases, it is the first report of OB/OP alpha-synuclein pathology in an unselected, population-based cohort. It should be noted, however, that this 85 + population may consist of superaged Finnish individuals that may not be directly comparable to the general population or to genetically more heterogeneous populations outside of Finland. In addition, the cohort has a large proportion of females, but was corrected for in statistical analyses.

In conclusion, our population based results showed that 41% of all cases had OB/OP alpha-synuclein pathology, and it was strongly associated with alpha-synuclein pathology elsewhere in the brain. Our results provide further support to the hypothesis of at least two divergent spreading routes of alpha-synuclein pathology.

## Supplementary Information

Below is the link to the electronic supplementary material.

Supplementary file1 (PDF 573 kb)
