# Supplementary material for: Alpha-synuclein pathology of olfactory bulbs/peduncles in the Vantaa85+ cohort exhibit two divergent patterns: a population-based study
Source: Acta Neuropathol. 2021 Sep 1;142(4):777–80. doi: 10.1007/s00401-021-02364-6 (PMC8423630; doi:10.1007/s00401-021-02364-6)
Supplement: Supplementary file 1 — Supplementary file1 (PDF 573 kb) [file 401_2021_2364_MOESM1_ESM.pdf]

Supplementary Information

**Alpha-synuclein pathology of olfactory bulbs/peduncles in the Vantaa85+ cohort exhibit two divergent patterns: a population based study**

Eloise H Kok[1]*, Sara Savola[1]*, Anna Raunio[1], Minna Oinas[2,3], Jarno Tuimala[2], Tuomo Polvikoski[4], Mia Kero[1], Karri Kaivola[5,6], Pentti J Tienari[5,6], Anders Paetau[1], Liisa Myllykangas[1]

*equal contribution

Affiliations

1 Department of Pathology, University of Helsinki, and HUS Diagnostic Center, Helsinki University Hospital, P.O. Box 21, 00014 Helsinki, Finland

2 Department of Pathology, University of Helsinki, P.O. Box 21, 00014 Helsinki, Finland

3 Division of Clinical Neuroscience and Rehabilitation, Department of Neurosurgery, Ophthalmology and Otorhinolaryngology, University Hospital of North-Norway N-9038 Tromso, Norway

4 Institute of Neuroscience, Newcastle University, Newcastle upon Tyne NE2 4HH, UK

5 Translational Immunology, Research Programs Unit, University of Helsinki, P.O.Box 63, 00014 Helsinki, Finland

6 Department of Neurology, Helsinki University Hospital, P.O. Box 63, 00014 Helsinki, Finland

**Corresponding author**

Liisa Myllykangas, Department of Pathology, University of Helsinki and HUS Diagnostic Center, Helsinki University Hospital, P.O Box 21, 00014 University of Helsinki, Finland. Tel. +358504482805, Fax. +358294126700

e-mail: liisa.myllykangas@helsinki.fi

## Materials and Methods

The Vantaa 85+ Study includes all individuals aged ≥ 85 years, living in the city of Vantaa, Finland, on 1 April 1991 (n=601). Neuropathological examination was possible in 304 subjects. The subjects or their relatives gave consent for the study. The study has been approved by local ethical committees.

OB/OP were collected separately from formaldehyde-fixed samples after several years of fixation specifically for this study. We categorised the results based on whether AON (bulbar AON and/or intrapeduncular AON regions according to Fig 1 in [4]) or peripheral regions were affected. Results therefore refer to LRP in the tertiary olfactory structures AON and secondary olfactory peripheral structures [10]. The samples were processed and placed into paraffin, after which standard protocols were used to immunohistochemically stain slides with alpha-synuclein 5G4 antibody (dilution 1:14000, concentration 0.2μg/ml) utilising a LabVision autostainer using standard protocols. A low pH target retrieval solution and 5 minutes of formic acid pretreatment were utilised. Compared to our previous study [9], we now utilised a more sensitive staining kit (Envision FLEX+ for mouse) and a new lot of the antibody, with different concentration, and thus a more diluted antibody was used in this study. Negative controls were applied by omitting the primary antibody, as well as replacing the primary antibody with IgG at the same concentration, otherwise following the protocol. These were performed in addition to the fact that approximately 60% of cases were negative and considered internal controls.

A total of 301 (99%) olfactory samples were available from the Vantaa 85+ cohort (n=304). A further 10 cases were removed because they were fragmented and did not contain clearly identifiable AON structures in HE staining, leaving 291 cases for the study. A total of 247 (85%) cases had identifiable peduncle and bulb tissue, whilst 43 (15%) had only peduncle and 1 (0.3%) had only bulb represented.

Cases were scored according to 'none' 'sparse' 'scattered' or 'dense' with scattered referring to 1-3 Lewy bodies at 100 x magnification and dense considered anything with more than 4 Lewy bodies at 100 x magnification. Lewy neurites were scored as 'none' 'sparse' 'scattered' or 'dense' through visual inspection and semiquantitative assessment. We used three categories of pathology staging to retain statistical power. Dot-like immunopositive structures were excluded, in accordance with the recommendations from [1]. Two assessors (EK and SS) both randomly examined all cases via microscope, without information on the clinical or pathological findings of the cases, and later compared results and on cases of disagreement, brought in a neuropathologist (LM) for final determination.

Predominance patterns were determined when individuals had at least one grade difference between peripheral and AON regions, or it was visibly possible to differentiate a preferential area. It was difficult to determine predominance in cases with modest pathology and thus we chose to only designate predominance in those with definite differences between regions, whilst the rest were regarded as unclassifiable.

CERAD score, Braak staging, and NIA RI AD protocols used have been previously described [7]. Methods for APOE genotyping have also been published earlier [5]. Dementia status was assessed by DSMIIII criteria and substantia nigra neuron loss as described in [6].

Statistical methods to assess the association between categorical variables was determined using Fisher's exact test. Sex and age adjusted p-values were generated by a likelihood ratio test comparing two penalised likelihood logistic regression models, with and without the variable of interest. The effect of our new data on the division into caudo-rostral and the amygdala-based LRP progression patterns was described using K-means clustering. The results are displayed as a mean strength of the LRP staining pathology for each anatomical region in each cluster. The optimal number of clusters for the K-means analysis was determined using the elbow method [2]. Statistical analyses were performed in SPSS for Windows v.27 and R 4.0.5 (R Core Team) [8].

Images were created by Microsoft Excel and R.

Supplementary **Table 1**

| | None | All positive | AON[#] predominant | Periphery[#] predominant |
|---|---|---|---|---|
| *n* | 172 | 119 | 79 | 21 |
| Women (%) | 84.3 | 80.7 | 77.2 | 90.5 |
| Mean age at death (years) | 92 | 93 | 93 | 92 |
| Age at death (*n*, %) | | | | |
| 85-89 | 47 (27.3) | 30 (25.2) | 20 (25.3) | 5 (23.8) |
| 90-94 | 84 (48.8) | 55 (46.2) | 35 (44.3) | 12 (57.1) |
| >95 | 41 (23.8) | 34 (28.6) | 24 (30.4) | 4 (19.0) |
| AON LRP (*n*, %) *a)****b)**** | | | | |
| none | **172 (100)** | **9 (7.6)** | **0** | **8 (38.1)** |
| sparse positivity | **0** | **30 (25.2)** | **1 (1.3)** | **12 (57.1)** |
| scattered§ | **0** | **22 (18.5)** | **20 (25.3)** | **1 (4.8)** |
| dense§ | **0** | **58 (48.7)** | **58 (73.4)** | **0** |
| Periphery LRP (*n*, %) *a)**** | | | | |
| none | **172 (100)** | **0** | 0 | 0 |
| sparse | **0** | **92 (77.3)** | 59 (74.7) | 15 (71.4) |
| scattered | **0** | **27 (22.7)** | 20 (25.3) | 6 (28.6) |
| dense | **0** | **0** | 0 | 0 |
| Braak NFT stage (*n*, %) *a)*** | | | | |
| 0-II | **58 (33.7)** | **29 (24.4)** | 18 (22.8) | 5 (23.8) |
| III-IV | **86 (50.0)** | **50 (42.0)** | 31 (39.2) | 7 (33.3) |
| V-VI | **28 (16.3)** | **40 (33.6)** | 30 (38.0) | 9 (42.9) |
| CERAD score (*n*, %) | | | | |
| None | 45 (26.2) | 24 (20.2) | 10 (12.7) | 4 (19.0) |
| Sparse | 21 (12.2) | 8 (6.7) | 4 (5.1) | 4 (19.0) |
| Moderate-frequent | 106 (61.6) | 87 (73.1) | 65 (82.3) | 13 (61.9) |
| NIA-RI (*n*, %) *a)*** | | | | |
| No | **34 (40.0)** | **16 (20.5)** | 8 (14.8) | 4 (28.6) |
| Yes | **51 (60.0)** | **62 (79.5)** | 46 (85.2) | 10 (71.4) |
| Substantia Nigra neuron loss (*n*, %) *a)****b)**** | | | | |
| None | **169 (98.8)** | **20 (16.8)** | **3 (3.8)** | **13 (61.9)** |
| Mild | **2 (1.2)** | **22 (18.5)** | **13 (16.5)** | **4 (19.0)** |
| Moderate | **0** | **19 (16.0)** | **12 (15.2)** | **3 (14.3)** |
| Severe | **0** | **41 (34.5)** | **35 (44.3)** | **1 (4.8)** |
| Very severe | **0** | **17 (14.3)** | **16 (20.3)** | **0** |
| Dementia status at death (*n*, %) *a)***b)*** | | | | |
| No | **71 (41.3)** | **30 (25.2)** | **13 (16.5)** | **9 (42.9)** |
| Yes | **101 (58.7)** | **89 (74.8)** | **66 (83.5)** | **12 (57.1)** |
| Age at dementia onset | 87 | 88 | 87 | 87 |
| Duration of dementia | 5.2 | 5.3 | 5.4 | 6.6 |
| APOE e4 (*n*, %) *a)** | | | | |
| No | **120 (73.2)** | **66 (59.5)** | 38 (51.4) | 14 (73.7) |
| Yes | **44 (26.8)** | **45 (40.5)** | 36 (48.6) | 5 (26.3) |
| DLB Consortium class. (*n*, %) *a)****b)**** | | | | |
| None | **157 (91.3)** | **15 (12.6)** | **0** | **13 (61.9)** |
| Non-classifiable | **11 (6.4)** | **0** | **0** | **0** |
| Brainstem | **4 (2.3)** | **13 (10.9)** | **2 (2.5)** | **5 (23.8)** |
| Amygdala-predominant | **0** | **10 (8.4)** | **7 (8.9)** | **1 (4.8)** |
| Limbic | **0** | **40 (33.6)** | **29 (36.7)** | **2 (9.5)** |
| Diffuse Neocortical | **0** | **41 (34.5)** | **41 (51.9)** | **0** |
| LRP progression based (*n*, %) *a)**** | | | | |
| Caudo-rostral | **15 (100)** | **65 (63.1)** | 44 (56.4) | 6 (75.0) |
| Amygdala-based | **0** | **38 (36.9)** | 34 (43.6) | 2 (25.0) |

§scattered = 1-3 Lewy bodies at 100x magnification; dense = >4 Lewy bodies at 100x magnification.
p-values were generated from likelihood ratio tests comparing two penalised likelihood logistic regression models, with and without the variable of interest (including age at death and gender), or Fisher exact tests.
* p≤0.05, ** p≤0.01, *** p≤0.001
[#] In 19 cases the predominance could not be determined
*a)* comparing none vs all positive OB/OP staining *b)* between predominance groups (AON vs peripheral)

Supplementary **Table 2**

Cases with positive OB/OP staining but no DLB consortium classification (n=15)

| Case | Braak | CERAD | OB/OP staining | AON LRP | Periph LRP | OB/OP Predominance | Gender | Age (y) |
|---|---|---|---|---|---|---|---|---|
| 1 | 3 | 1 | Both | sparse | sparse | Peripheral | F | 93 |
| 2 | 3 | 0 | Both | sparse | sparse | Peripheral | F | 87 |
| 3 | 5 | 2 | Both | sparse | scattered | Peripheral | F | 87 |
| 4 | 6 | 2 | Periphery only | none | sparse | Peripheral | F | 87 |
| 5 | 5 | 2 | Both | sparse | sparse | Peripheral | F | 92 |
| 6 | 5 | 3 | Periphery only | none | sparse | Peripheral | F | 91 |
| 7 | 6 | 2 | Both | sparse | sparse | Peripheral | F | 92 |
| 8 | 5 | 2 | Both | sparse | sparse | Peripheral | F | 101 |
| 9 | 3 | 2 | Periphery only | none | sparse | Peripheral | F | 92 |
| 10 | 1 | 1 | Periphery only | none | sparse | Peripheral | F | 97 |
| 11 | 2 | 3 | Periphery only | none | sparse | Peripheral | F | 93 |
| 12 | 6 | 3 | Both | sparse | sparse | Peripheral | M | 93 |
| 13 | 3 | 0 | Both | sparse | scattered | Peripheral | F | 97 |
| 14 | 5 | 2 | Both | sparse | sparse | N/A | F | 94 |
| 15 | 4 | 0 | Periphery only | none | sparse | N/A | F | 94 |

Supplementary **Fig. 1** Olfactory Bulb/Penduncle predominance according to DLB Consortium classification [3].

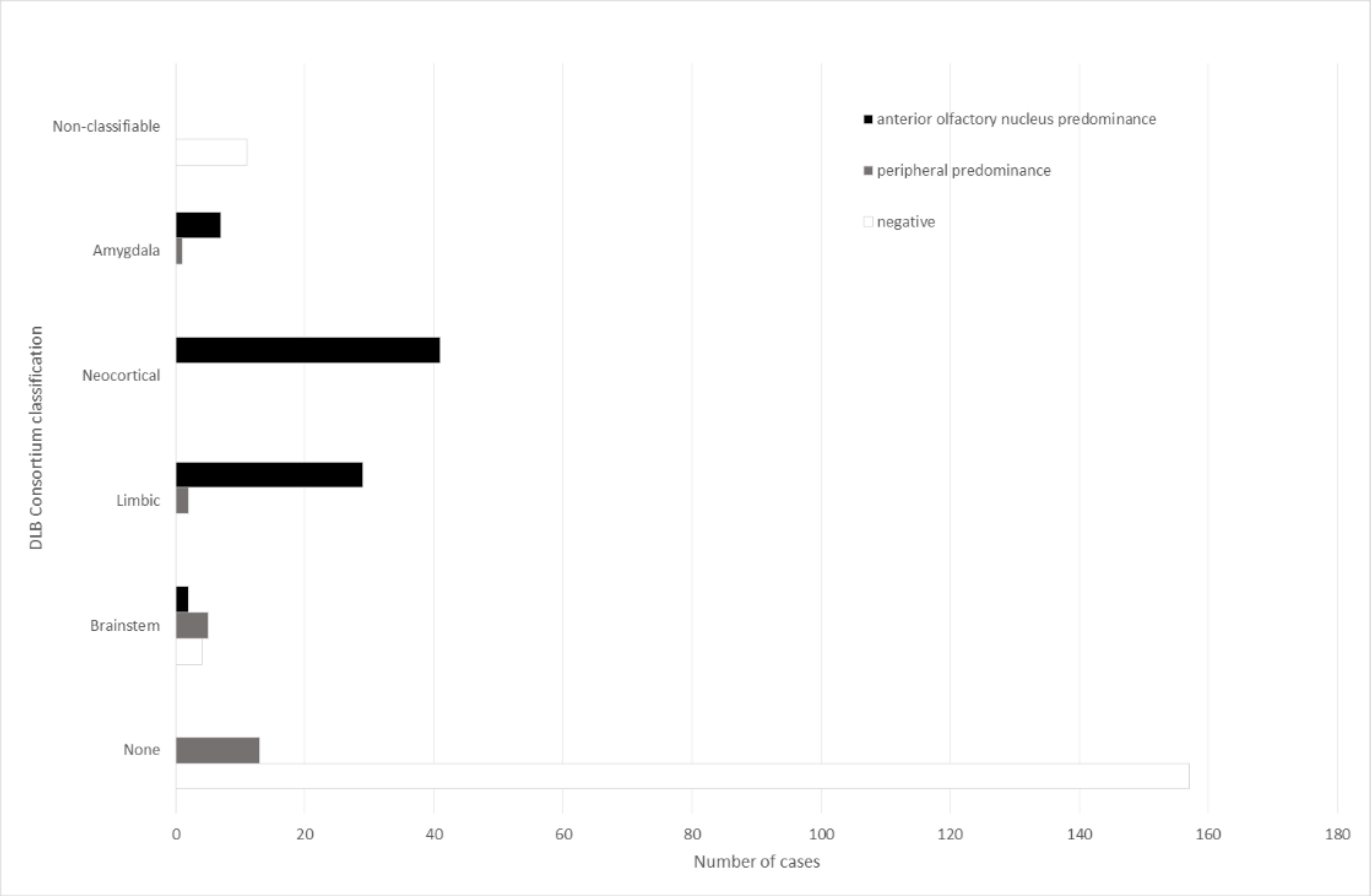

Supplementary **Fig. 2** Classification of cases by progression pattern of LRP as determined by K-means cluster analysis.

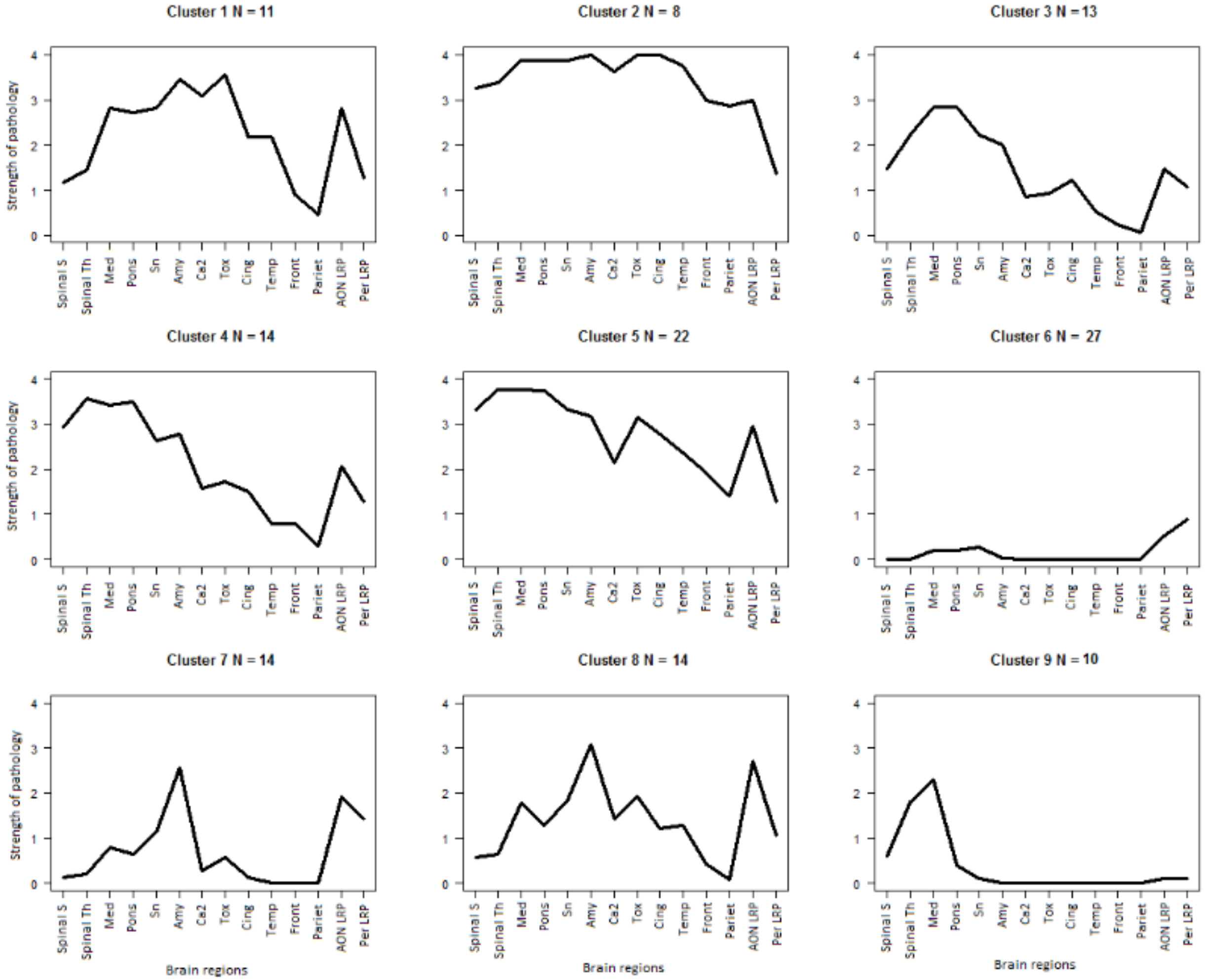


On the y-axis is the semi-quantitative LRP score (0-4 all brain regions, except OB – 0-3). On the x-axis are the different CNS regions: Spinal S – sacral spinal cord, Spinal Th – thoracic spinal cord, Med – medulla, Pons – pons, Sn – substantia nigra, Amy – amygdala, Ca2 – CA2 of hippocampus, Tox – transentorhinal cortex of hippocampus, Cing – cingulate cortex, Temp – temporal cortex, Front – frontal cortex, Pariet – parietal cortex, AON LRP – olfactory bulb/peduncle anterior olfactory nucleus LRP, Per LRP –olfactory bulb/peduncle periphery LRP.

Supplementary **Fig. 3** Olfactory Bulb/Peduncle predominance according to Substantia nigra neuronal loss.

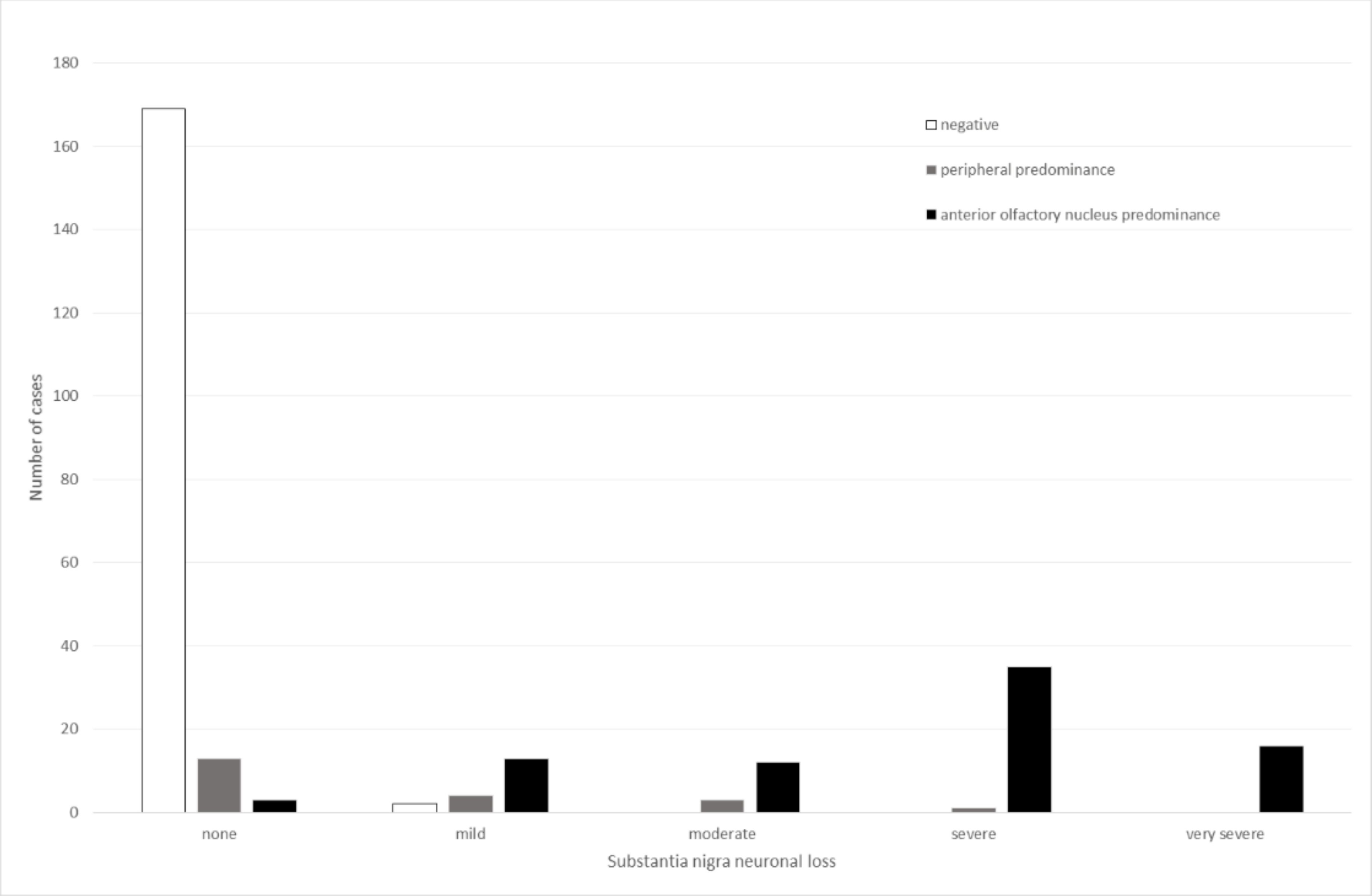

## References


1 Attems J, Toledo JB, Walker L, Gelpi E, Gentleman S, Halliday G, et al (2021) Neuropathological consensus criteria for the evaluation of Lewy pathology in post-mortem brains: a multi-centre study. Acta neuropath 141(2):159–72. https://doi.org/10.1007/s00401-020-02255-2

2 Ketchen DJ, Shook CL (1996) The application of cluster analysis in strategic management research: an analysis and critique. Strateg Manag J 17:441–458. https://doi.org/10.1002/(sici)1097-0266(199606)17:6%3c441:Aid-smj819%3e3.0.Co;2-g

3 McKeith IG, Boeve BF, Dickson DW, Halliday G, Taylor JP, Weintraub D et al (2017) Diagnosis and management of dementia with Lewy bodies: Fourth consensus report of the DLB Consortium. Neurology 89(1), 88–100. https://doi.org/10.1212/WNL.0000000000004058

4 Murray HC, Dieriks BV, Swanson MEV, Anekal PV, Turner C, Faull RLM, et al. The unfolded protein response is activated in the olfactory system in Alzheimer’s disease. Acta neuropathologica communications. 2020;8(1):109–109.

5 Myllykangas L, Polvikoski T, Sulkava R, Verkkoniemi A, Crook R, Tienari PJ et al (1999) Genetic association of alpha2-macroglobulin with Alzheimer's disease in a Finnish elderly population. Ann Neurol 46(3), 382–390.  PMID: 10482269.

6 Oinas M, Polvikoski T, Sulkava R, Myllykangas L, Juva K, Notkola IL et al (2009) Neuropathologic findings of dementia with lewy bodies (DLB) in a population-based Vantaa 85+ study. J Alzheimers Dis 18(3), 677–689. https://doi.org/10.3233/JAD-2009-1169

7 Peuralinna T, Tanskanen M, Mäkelä M, Polvikoski T, Paetau A, Kalimo H et al (2011) APOE and AβPP gene variation in cortical and cerebrovascular amyloid-β pathology and Alzheimer's disease: a population-based analysis. J Alzheimers Dis 26(2), 377–385. https://doi.org/10.3233/JAD-2011-102049

8 R Core Team 2020 R: A language and environment for statistical computing. R Foundation for Statistical Computing, Vienna, Austria. URL https://www.R-project.org/

9 Raunio A, Kaivola K, Tuimala J, Kero M, Oinas M, Polvikoski T et al (2019) Lewy-related pathology exhibits two anatomically and genetically distinct progression patterns: a population-based study of Finns aged 85. Acta Neuropathol 138(5), 771–782. https://doi.org/10.1007/s00401-019-02071-3

10 Sengoku R, Saito Y, Ikemura M, Hatsuta H, Sakiyama Y, Kanemaru K et al (2008) Incidence and extent of Lewy body-related alpha-synucleinopathy in aging human olfactory bulb. J Neuropathol Exp Neurol 67(11), 1072–1083. https://doi.org/10.1097/NEN.0b013e31818b4126
